# Regulatory roles of LINE-1-encoded reverse transcriptase in cancer onset and progression

**DOI:** 10.18632/oncotarget.2504

**Published:** 2014-10-04

**Authors:** Ilaria Sciamanna, Alberto Gualtieri, Pier Vincenzo Piazza, Corrado Spadafora

**Affiliations:** ^1^ Istituto Superiore di Sanità, Rome, Italy; ^2^ NeuroCentre Magendie, INSERM U862, Univ Bdx2, Bordeaux, France

**Keywords:** Retrotransposon, reverse transcriptase, inhibitor, non coding RNA, cancer therapy

## Abstract

LINE-1 retrotransposons encode the reverse transcriptase (RT) enzyme, required for their own mobility, the expression of which is inhibited in differentiated tissues while being active in tumors. Experimental evidence indicate that the inhibition of LINE-1-derived RT restores differentiation in cancer cells, inhibits tumor progression and yields globally reprogrammed transcription profiles. Newly emerging data suggest that LINE-1-encoded RT modulates the biogenesis of miRNAs, by governing the balance between the production of regulatory double-stranded RNAs and RNA:DNA hybrid molecules, with a direct impact on global gene expression. Abnormally high RT activity unbalances the transcriptome in cancer cells, while RT inhibition restores ‘normal’ miRNA profiles and their regulatory networks. This RT-dependent mechanism can target the myriad of transcripts - both coding and non-coding, sense and antisense - in eukaryotic transcriptomes, with a profound impact on cell fates. LINE-1-encoded RT emerges therefore as a key regulator of a previously unrecognized mechanism in tumorigenesis

## INTRODUCTION

LINE-1 elements are the largest family of human retrotransposons, mobile genetic elements that move in the human genome via an RNA intermediate. The LINE-1 family comprises about 500.000 copies, collectively accounting for as much as 17% of the human genome [1]. Each LINE-1 copy encodes a bicistronic RNA transcript which is translated into a 40 kDa RNA-binding protein (ORF-1) and a 150 kDa protein (ORF-2), the latter endowed with endonuclease and reverse transcriptase (RT) activities [2]. As such, RT is the most highly repeated protein-coding sequence in the genome of higher eukaryots and an essential component of the retrotransposition machinery, required not only for the mobilization of its own coding elements, but also for other non-autonomous retrotransposons, such as Alu and SVA [3].

At the origin of the discovery of mobile elements is their ability to produce phenotypic variations by integrating at mutliple genomic sites, hence interrupting the physical continuity and functional integrity of genes, which was historically recognized even before our understanding of gene organization and function. In the case of LINE-1, however, only a minor fraction (about 80–100) of all copies present in the human genome are full-length and retrotranspositionally competent [4], whereas the vast majority of genomic LINE-1 copies are truncated at their 5’ end and thus non-mobile [5], yet still transcriptionally competent: this implies that LINE-1 elements have a higher potential for producing a proficient RT enzyme (encoded by the ORF-2 present in all copies) than for retrotransposition (of which only the full-length elements with an intact 5’ end are capable). This indicates that RT production is not necessarily coupled with retroelement mobility, and highlights the notion that the transcriptional capability of the considerable high number of genomic elements provides cells with a potentially large source of RT activity.

### Retroviral and retroelement-derived reverse transcriptase

The groundbreaking discovery of an RT activity encoded by infective retroviruses [6, 7] has revolutionized our understanding of genome function, because it showed the existence of an unanticipated flow of genetic information, from RNA to DNA, in contrast with the central dogma of molecular biology - which considered DNA to RNA as the only possible direction. Howard Temin first predicted a functional role for RT, both in physiological differentiation, as in embryogenesis, and in its pathological loss, as in cancer [8]. Temin's visionary prediction was fulfilled, in some way, by the discovery that non-infected cells are also endowed with an endogenous RT that can act on the genetic information stored in nuclei and provide a source of continuous genomic variability. A considerable body of evidence after Temin's discovery has shown that the expression of endogenous RT is itself developmentally modulated and is implicated in a broad spectrum of pathological and physiological settings. Indeed, non-pathological differentiated tissues contain low levels of RT activity, if at all, while high RT activity is typically found in embryos and embryonic tissues [9].

Besides embryonic tissues, the endogenous RT is generally abundant in cells characterized by a low differentiation levels and a high proliferation rate, such as transformed cells [10], consistent with the observation that retroelements are mobilized in many pathologies, including tumors [11, 12]. Thus, undifferentiated or dedifferentiated cells and tissues with a highly proliferating potential constitute permissive systems for RT expression and retrotransposition activity, while differentiated quiescent cells offer less favourable contexts [13].

While the RTs of infective retroviruses, of clear clinical relevance to infected cells, have been intensely investigated [reviewed in 14], the endogenous RT has received lower attention, in spite of the many clues that overtly suggested a potential implication in fundamental physiological and pathological processes.

Only in the last decade have roles of the LINE-1-encoded RT been recognized, both in embryogenesis and in tumorigenesis [respectively examined in 9 and 10]. The RT has emerged as a key regulator of both these processes, in parallel with the increasingly recognized contribution of transposable elements to genome-wide regulatory networks [15]. Recent evidence indicates however that retroelement mobilization reflects only part of the roles of RT in the retrotransposition machinery. Here we review evidence linking the endogenous LINE-1-encoded RT to tumorigenesis and propose a model for a previously unrecognized regulatory role in the genesis and progression of cancer. To define the newly emerging role of RT, in the next section we will briefly recall some essential aspects of the eukaryotic transcriptome and its links with retrotransposon networks.

### Genomes are pervasively transcribed on both strands: implications in cancer

The historical legacy that the eukaryotic transcriptome is constituted by messenger RNA (mRNA), transcribed from protein-coding genes, and by the non-coding ribosomal RNAs (rRNA) and transfer RNAs (tRNA), has radically changed in recent years. It is now well-established that the vast majority of eukaryotic genomes are pervasively transcribed [16].

The advent of next generation sequencing technologies led to the unexpected discovery of varieties of non-coding RNAs (ncRNAs) [reviewed in 17, 18, 19]. ncRNAs are grouped in two major classes, small RNAs (sncRNAs) < 200 bp, typically unstable, and long RNAs (lncRNAs) ranging from > 200 bp to 100 kb [20] more stable, transcribed on either or both of the DNA strands and classified according to distinctive sequence features [17, 19, 21). The discovery of microRNAs (miRNAs) and of naturally occurring small interfering RNAs (siRNAs) [reviewed in 22] provided early evidence that not only the transcriptional landscape is of higher complexity than ever thought, but also that these RNAs have regulatory roles. lncRNAs are integral components of the mammalian transcriptome [17, 19, 21, 23] and constitute a highly heterogeneous class of thousands of polymerase II-transcribed RNA species, polyadenylated, spliced, mostly localized in the nucleus [reviewed in 24]. The evidence that the vast majority of genomic transcription is non-coding, whereas only less than 2% is transcribed in protein-coding mRNAs [16], suggests that the former cannot be dismissed as mere functionless transcriptional “noise”, but may have functional roles.

Another recently identified component of the transcriptome is composed of natural antisense non-coding RNA transcripts (NATs) from both protein-coding and non-coding genes [25, 26]. Antisense transcripts are widespreadly produced across the genome of various species [27, 28]. They represent a pervasive phenomenon, accounting for about 50–70% of annotated human coding sequences having sense partners, including genes with relevant developmental functions [29]. They are on average 10-fold less abundant than sense expression and preferentially stored in nuclei [reviewed in 30, 31]. Interestingly, antisense transcription occurs nonrandomly across the genome [32] and is concentrated at preferential “hot spots” overlapping both ends of coding genes [33, 34]. Several NATs have a regulatory role on gene expression [35]. Together with sense lncRNAs, NATs are components of complex genome-wide regulatory networks that finely tune the genome expression, with roles in tumorigenesis, differentiation and development [reviewed in 29, 31, 36, 37].

A vast body of data implicate ncRNA classes, both sense and antisense transcripts, in tumorigenesis [reviewed by 38, 27], a context in which the transcriptome is profoundly affected by altered genome methylation [39, 40]. miRNAs were the first class of ncRNAs to be implicated in cancer formation and spreading: miRNAs can act as oncogenes or tumor suppressors [41, 42], are frequently located within cancer-associated genomic regions [43] and show significantly altered expression profiles in human cancers, a dysregulation caused by both epigenetic and genetic changes [38], often sufficient to induce oncogenesis [44, 45]. Long non-coding RNAs (lncRNAs) expression [46, 47] is highly tissue-specific compared with coding genes, a finding consistent with the hypothesis that lncRNAs contribute to confer target specificity to regulatory networks [48, 49]. Serial analysis of gene expression libraries (SAGE) indicate that the tissue-specificity of lncRNA expression is altered in many cancer types [50], suggesting roles in tumorigenesis. T-UCRs are lncRNAs transcribed from ultra-conserved regions (UCRs) [51] acting as possible developmental enhancers in mammalian genomes [52] and aberrantly expressed in a variety of human cancers [53, 54, 55].

### ncRNAs and transposable elements: a long-lasting relationship

Remarkably, ncRNAs and transposable elements (TEs) share many biogenetic, functional and structural aspects [56, 57, reviewed in 58]:

- First, a high proportion of miRNAs originates from TE families, including DNA transposons, LTR-containing retrotransposons, LINE-1 and SINE elements [58, 59].
- Second, TE sequences are embedded in about three-quarters of all mature long non-coding (lnc) RNA transcripts, while being virtually absent from protein-coding exons, and account for about 30–42% of total human lncRNA sequences [56, 57]. Interestingly, TEs - particularly LTR-containing ERVs - target preferential positions and orientations within lncRNAs; they are frequently associated with transcription starting sites (TSS), and hence may have roles in regulation of lncRNA transcription. Importantly, a relevant regulatory role has been assigned to HERV- H-containing lncRNAs expressed in embryonic stem cells (ESCs) [56, 57] and to other lncRNAs enriched in LTRs, the expression of which is implicated in pluripotency of ESCs [60, 61].
- Additionally, retrotransposition events can generate thousands of pseudogenes, which also have global regulatory roles [62]. The “competing endogenous RNA” (ceRNA) hypothesis [63] highlights the regulatory role played, among others, by pseudogenes generated via mRNA retrotransposition. In the ceRNA hypothesis, which takes into account the variety of targets for each miRNA and the variety of miRNAs capable of acting on a common target, cross-talks are generated between distinct regulatory RNAs; pseudogenes may “sequester” specific miRNAs and hence modulate their actual availability as functional regulatory molecules. In this framework, pseudogene transcripts, mRNAs and lncRNAs constitute regulatory networks, the “communication” of which is mediated by a limited pool of miRNAs [62].

Overall, the phenomena briefly outlined above entail different levels of control (e.g., transcriptional in the case of some inserted retrocopies, post-transcriptional in cases in which retrotransposons originate regulatory RNAs or, on the contrary, block their function); their common reverse transcription-dependent origin indicates the many ways through which RT can globally shape genome functions. Collectively therefore these data indicate a broad reach of TEs in shaping the transcriptome of ncRNAs and influencing their regulatory role and tissue specificity.

### Retrotransposons and the endogenous RT in tumorigenesis

The notion that expression of retroelements increases in tumors, while being low in normal tissues, is consistent with recent findings that proteins encoded by the LINE-1 bicistronic open reading frames, i.e. ORF1p and ORF2p, are abundant in a variety of cancers [64], breast [65; 66], gastric [67] and pediatric germ cell tumors [68], but not in their healthy tissue counterparts.

**Figure 1:**
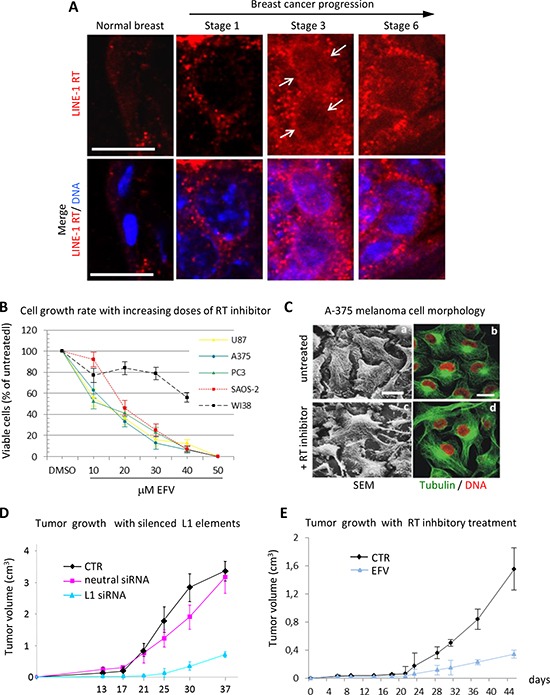
RT inhibition recapitulates the global reprogramming of cancer cell phenotypes observed with LINE-1 element silencing **(A)** Distribution of RT protein (depicted in the top panels in red and, below, in merged confocal images with Hoechst-stained nuclei) during murine mammary cancer progression. Both the abundance and the perinuclear accumulation (arrowed) of RT increase in progressive cancer stages (numbered 1–6; for the description of staging criteria see [69]). Bars, 10 micrometers. **(B)** The RT inhibitor EFV inhibits proliferation of transformed but not of normal cells. The curves represent the percentage of cells after four days of culture with increasing concentrations of EFV. **(C)** EFV induces morphological differentiation of A-375 melanoma cells. Scanning electron microscopy (left panels) and confocal microscopy (right panels) depict the cytoskeletal reorganization and the elongated morphology induced by EFV (c-d) compared to the undifferentiated shape of untreated cells **(a-b)**. **(D)** Reduced tumorigenicity of A-375 melanoma cells interfered for LINE-1 in animal models. Tumor progression was monitored in nude mice inoculated with A-375 cells either untreated, or stably interfered with a neutral, or with LINE-1 (pS-L1i, indicated here as L1-specific siRNAs. Curves show tumor growth (average volume measured in groups of five animals) at the indicated times after melanoma cell inoculation. **(E)** EFV treatment reduces human A-375 tumor growth in nude mice. A-375 melanoma cells were xenografted in nude mice as for panel D and tumor progression was monitored in animals, either untreated or treated with EFV starting 1 day after cell inoculation. Curves show the mean tumor size in groups of five animals at the indicated times. Experimental details can be found in [69] (panel A), [87] (B), [78] (C and E), [79] (D)

In agreement with these studies, using a specific RT-targeted monoclonal antibody we have depicted a quantitative increase of LINE-1-ORF2p proteins in progressive breast cancer stages in a cancer-prone transgenic mouse model [69]. Exemplifying immunofluorescence panels in Fig. 1A illustrate this increase in progressively advanced stages of breast cancer. Cancer samples withdrawn from mice at regular intervals after birth were staged (1 to 6 ) according to several parameters (e.g. expression of epidermal growth factor receptor (ERB2), down-regulation of the estrogen receptor (ER) and others; see [69] for detailed description). We found that both LINE-1 and SINEB1 retroelements undergo progressive copy number amplification in advancing cancer stages, indicating that the activation of the retrotransposon machinery yields not only increased expression, but also an increase in the content of retroelements in the cancer cell genome [69].

The activation of the retrotransposition machinery can yield extensive genomic insertions, typical of human cancers: indeed, hundreds of novel cancer-specific retrotranspositions have been mapped in genomes from lung [70], colon [71, 72], prostate [71], ovarian [71] and liver [73] carcinomas. While these data confirm that tumors offer a highly permissive environment for retrotransposition, they do not indicate whether these insertions are “passenger” (irrelevant to the onset of oncogenesis) or “driver” mutations with a causative potential [74], favouring the emergence of a typically altered “cancer genome” [75].

Compelling evidence indicate that retrotransposition events exert a profound impact on genome function and expression, with a crucial role of the endogenous RT. LINE-1-encoded RT is an essential mechanistic component for the mobility of retrotransposons, usually not thought to play any other role beyond retrotransposition. Intrigued by the evidence implicating retrotransposition in the physiological state of cells and tissues, we sought to directly assess the role of RT in tumorigenesis using two experimental approaches. In the first one, RT was pharmacologically inhibited in cancer cell lines using nonnucleoside inhibitors widely employed in AIDS treatment, e.g. nevirapine or efavirenz (EFV) [76, 77, 78]; in the second one, we used the RNA interference (RNAi) methodology to down-regulate the expression of full-length RT-encoding LINE-1 elements, the major source of RT activity in human cells [4], in cancer cell lines [78, 79]. Both approaches yielded consistent responses: first, RT inhibition caused a reduced cell proliferation rate in cancer cells transformed but not in normal cells (e.g. WI38 fibroblasts) (Fig. 1B); second, cells assumed a differentiated phenotype (exemplifying panels from A375 melanoma cultures are depicted in Fig.1C). The changes in cell functional morphology (such as seen in Fig. 1C), including the appearance of dendritic-like extensions and increased adhesion to the plate surface, distinctive of differentiating melanoma (see [78] for an in-depth characterization), are accompanied by global alterations of the transcriptome of coding and non-coding sequences, as will be seen below. Several laboratories have independently confirmed these conclusions in studies of various human tumorigenic cell lines treated with RT inhibitors, both of the nonnucleoside [80, 81, 82] and the nucleoside type [83, 84, 85].

Importantly, RNAi-mediated LINE-1 down-regulation in transformed cells drastically reduced their tumorigenic potential in nude mice cancer xenografts (Fig.1D) [79]. Remarkably, RT inhibitors exert a very similar, powerful anti-cancer effect *in vivo*; Fig. 1E shows that EFV treatment of mice xenografted with human melanoma cells arrested, or significantly reduced, tumor progression [78]. The proliferation inhibitory and differentiation-promoting effects associated with RT inhibition, both in cell cultures and in animal models *in vivo*, are reversible: on discontinuation of the treatment, tumor cells returned to their original rate of proliferation and dedifferentiated phenotype [78]. Thus, nonnucleoside inhibitors reversibly prevent the activation of pathways orchestrated by LINE-1-encoded RT in cell proliferation and differentiation and, ultimately, in cancer growth in vivo.

In contrast, no significant effect was associated with down-regulation of HERV elements, which encode a related yet distinct RT [79]. On the other hand, the inhibition of the telomerase reverse transcriptase (h-TERT) has an effect, yet that is clearly distinct from that of LINE-1 RT: first, h-TERT is sensitive to specifically targeted inhibitors but not to nonnucleoside RT inhibitors nevirapine or efavirenz [86, 76, our unpublished results]; second, TERT inhibition results in a slow-proceeding kinetics of cancer growth inhibition, requiring several rounds of cell division, consistent with effects exerted via telomere metabolism. The growth suppressive effects associated with LINE-1 RT inhibition are instead already appreciated within the first 48–72 hours of inhibitory drugs. Thus, the anticancer effect of nonnucleoside inhibitors are selectively exerted via inhibition of the LINE-1-encoded RT.

The similarity of the tumor suppressive effects seen in cultures either treated with RT inhibitors, or silenced for LINE-1 expression, suggests that the inhibition of RT activity is sufficient to recapitulate the effects of silencing the LINE-1 active elements. We will return to this point in a following paragraph. The data summarized thus far suggest that both the RT protein and the RT-encoding genes can be regarded as cancer therapeutic targets and imply that RT inhibitors can be effectively used in a non-cytotoxic differentiation therapy of cancer.

### LINE-1-encoded RT globally regulates genome expression

To clarify the RT-dependent mechanism implicated in cancer, we carried out a global expression profile analysis of protein-coding mRNAs, miRNAs and T-UCRs, in A-375 human melanoma cells in their native state and after RT inhibitory treatment with EFV [87]. This revealed that RT inhibtion yields an extensive reprogramming, including up- or down-regulation, of the transcription profiles of coding and non-coding RNAs (Box 1).

**Box 1:** RNA classes up- or down-modulated by (EFV)-dependent RT inhibition in A-375 melanoma cells

RNA class	Total examined (n)	EFV-modulated expression (up or down)	
		(n)	(%)
Protein-coding	14.000	854	6, 1
miRNAs	726	35	4, 8
UCRs	481	52	10, 8

Consistent with these findings in melanoma cells, a recent study independently reported that LINE-1 downregulation in breast cancer cells alters the expression of miRNA subpopulations [88].

In our study of RT-inhibited melanoma cells, hints to understand how the RT-dependent mechanism might operate came from a closer inspection of the EFV- down-regulated populations of miRNAs and UCRs: first, we noticed that a fraction was associated with over-represented flanking Alu elements, a large proportion of which is made up of closely spaced pairs of inverted Alu repeats (i.e. arranged in opposite orientation). Moreover, most RT-sensitive miRNAs and UCRs target oncogenes or tumor suppressors located in fragile sites or cancer-associated regions. Notably, RT inhibition reversed the expression profile of a sub-group of ten miRNAs, classified as metastamiRs, with crucial roles in tumor invasion and metastasis [89]: those that are up-regulated in cancer cells were found to be down-regulated in RT-inhibited cells and viceversa.

Crucial to interpret these data was the identification, through buoyant density gradient centrifugation, of particular molecules, containing LINE-1- and Alu sequences and having the buoyant density of RNA:DNA hybrids, present in melanoma and prostate carcinoma cells, but not in non-transformed cells [87]. The hybrid molecules were heterogeneous in size (from 40 nt to roughly 1 kb) and their presence was abrogated in cancer cells treated with RT inhibitor: this finding indicates therefore that their genesis and maintenance is dependent on RT function, and, furthermore, suggests functional links connecting the RT activity, the regulation of specific transcript classes and the transition of cells between normal and cancer state.

### A model for a novel RT-dependent tumor-promoting mechanism

What follows is a speculative attempt to integrate the findings summarized above in a comprehensive model that may account for the global role of LINE-1-encoded RT in cancer and help rationalize the anti-cancer effect of RT inhibitors. The model, building on the observed reprogramming of retroelement-containing miRNAs after RT inhibition [87], might extend to virtually all classes of RNAs - coding and non-coding, sense and antisense - and encompass the lines of evidence discussed above:

- RT activity is abundant in tumor cells and is absent, or poorly expressed, in their healthy counterpart of same histological origin;
- RNA:DNA hybrid molecules are detected in cancer but not in healthy cells, and disappear in RT-inhibited, phenotypically “cured” cancer cells: this establishes a direct link between high RT activity and formation of RNA:DNA hybrids in cancer cells;
- the formation of RNA:DNA hybrids is associated with an extensive reprogramming of the expression profiles of various RNA classes; this suggests that the hybrid molecules affect the normal function of such RNAs, by either altering their regulatory roles or blocking their translation.

**Figure 2:**
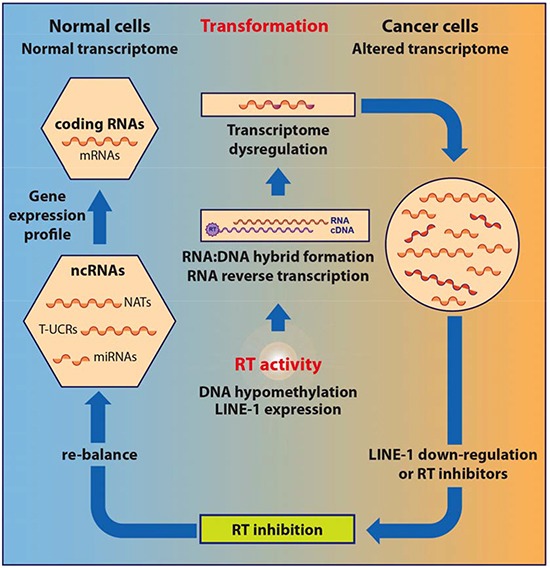
Model for RT-mediated control of the transcriptome in cancer cells Coding and non-coding RNAs constituting the transcriptome of normal cells (left side of the figure) are intercepted by the highly expressed RT in transformed cells (central part of the figure), reverse-transcribed and converted in RNA:DNA hybrid structures, with the ensuing transcriptome dysregulation in cancer cells (right side of the figure). Inhibition of RT activity in cancer cells restores the normal regulatory RNA profile and reverts the cell phenotype from cancer to normal.

Fig. 2 illustrates the proposed mechanism for the role of LINE-1-encoded RT in tumorigenesis. LINE-derived RT activity is up-regulated in early tumorigenesis [69], associated with altered patterns of DNA methylation [39, 40] and activation of LINE-1 retrotransposon expression. The overproduced RT can “intercept” RNA transcripts and abundantly reverse-transcribe them, with an increased production of RNA:DNA hybrid molecules [87]. We hypothesize that this is functionally equivalent to “sequestering” RNA templates for the formation of regulatory dsRNAs, with an ensuing decrease in the production of small regulatory RNAs, which ultimately compromises the expression profile of protein-coding genes. In this hypothetical model, the RT “subtracts” RNA strands and renders them unavailable for dsRNA formation, with a corresponding increase in RNA:DNA hybrid molecules. Consistent with this view, the biogenesis of LINE-1-derived miRNAs [90] and siRNAs [91] is globally reduced in cancer (i.e., high RT environment, abundant RNA:DNA hybrids) compared with normal (low RT activity, no or negligible formation of RNA:DNA hybrids) cells.

Small RNAs dysregulation in cancer cells is associated with a global re-tuning of the transcriptome, causing in turn the loss of differentiation, uncontrolled proliferation, altered DNA and chromatin epigenetic marks, typical of tumor cells. The down-regulation of miRNAs in cancer cells shares striking similarities with the suppression of miRNA functions in early embryos [92], a naturally occurring phenomenon correlated with three concomitant events:

- massive reduction of the overall methyl-cytosine content in the embryonic genome [93],
- bursts of retrotransposon expression [94], and
- increased activity of embryonic RT activity [95].

Deep profiling of transcriptomes in human and murine stem cells has recently identified a class of retroelement-derived transcripts specifically involved in the maintenance of pluripotency [96], confirming the regulatory role of ncRNAs of retrotransposon origin.

The regulatory role of RT may not be solely exerted via miRNAs; indeed, its reach includes UCR-lncRNAs as well [87]. It is reasonable to believe that RT-dependent regulatory networks can extend their effects to protein-coding mRNAs; the incorporation of the latter into RNA:DNA hybrid structures would constitute a major hurdle to proper translation, with direct implications over the cell fate. In support of this hypothesis is the recent finding that LINE-1-containing ribonucleoprotein particles comprise not only retrotransposon RNA, as expected, but also a variety of polymerase III- and II- transcribed RNAs, among which many annotated mRNAs [97]; this suggests that a large variety of RNAs might be taken up by the RT-dependent machinery, used as reverse transcription templates and rendered unavailable to the translation machinery. NATs are other potential candidates for reverse transcription: in that case, their functional inactivation in RNA:DNA hybrids would induce alterations in expression of specific genes, by preventing either the pairing of antisense complementary to coding sense transcripts, or the pairing with sense sequences located in 5’-UTR regulatory hot spots.

The pharmacological inhibition of RT in cancer cells prevents the formation of RNA:DNA hybrids; that coincides with the re-establishment of a non-pathological profile of regulatory RNAs, normalization of the transcription landscape and re-differentiation of cellular phenotype [87]. The RT-dependent RNA:DNA molecules present in cancer cells are as yet only partly characterized, though LINE-1 and SINE sequences were identified by PCR assays [87]. RT inhibitors that abolish the hybrid molecules concomitantly alter the expression profiles of miRNAs, lncRNAs and coding mRNAs, indicating therefore a circumstantial link between the RNA:DNA hybrid molecules on the one hand, and the production of regulatory RNAs on the other hand. More sequencing work will be required to thoroughly assess the identity of the hybrid structures and directly ascertain the mechanism proposed to lead from the inhibition of the LINE-1-encoded RT to the reprogramming of regulatory RNA profiles.

The highly complex mammalian transcriptome offers in principle countless RNA transcripts as potential substrates for reverse transcription, potentially expanding the influence of LINE-1-encoded RT to a large repertoire of cellular functions and processes. The effect of RT can therefore be further modulated under various types of stress, as stress is a well-known activator of LINE-1 expression [98]. On the whole, the LINE-1-encoded RT-dependent mechanism emerges as a global tumor-promoting system, impacting on regulator of the global transcriptome in the transition from normal to transformed cell states, and hence a promising target in cancer therapy.

A thought-provoking fact in this context is the total refractoriness of naked-mole rats to develop cancer [99]: strikingly, the genome of these rats has an unusually low content in retroelements (25%, compared to 40% and 45% of murine and human genomes, respectively) and, most importantly, none of their transposable element is viable [100]. It is tempting to attribute the amazing privilege of cancer resistance to the inactive state of the RT-mediated machinery.

### Genetics, epigenetics and the reversibility of cancer phenotypes

Retrotransposon-bearing genomes harbor a continuously reshaping source of genetic and epigenetic information entangled in their regulatory networks, which generate a broad repertoire of cellular phenotypes. The overall expression of this system is modulated during development, is tissue-specific and organized in highly dynamic pathways, sensitive to structural alterations, responsive to environmental stressors and, under certain conditions, reversible. Taking these elements into account, it should not be surprising that cell differentiation is a reversible phenomenon that can be reprogrammed by introducing transcription factors, or by nuclear transfer, into pluripotent/totipotent cells [reviewed in 101, 102]. These groundbreaking findings, while bridging the conceptual gaps between differentiated cells and their undifferentiated precursors, strongly support the view that cellular phenotypes are transient conditions with the potential to mutually convert into one another. Cancer cells may be viewed as paradigmatic examples of such reversible states, originating either from a de-differentiating process occurring in differentiated cells, or as the products of aberrant stem cell differentiation. Experimentally, transformed phenotypes can revert back to “normal” on exposure to differentiating conditions [103], and tumorigenic lesions can be epigenetically erased, at least to some extent, upon nuclear transfer reprogramming [104, 105]. Conversion to a differentiated phenotype can occur even in the presence (and in spite) of DNA alterations, because epigenetic changes can bypass the genetic alterations and reprogram gene expression, suggesting the conclusion that, overall, epigenetics wins over genetics [106]. The concept of phenotype reversibility has important clinical implications for the development of a non-cytotoxic differentiation cancer therapy [107].

The role of LINE-1-encoded RT (and anti-RT treatment) fit well into the epigenetic landscape, to the extent to which cancer-permissive or -repressive conditions are induced in cells by activating or inhibiting RT, respectively. As recalled above, RT inhibition alone is sufficient to stop tumor progression, reduce the tumorigenic potential of cancer cells and restore the normal cellular phenotype, yet this acquired differentiated state is only stable as long as the cells are under RT inhibition. On discontinuation of the RT inhibitory treatment, cancer cells resume their transformed phenotype and tumor progression is resumed in animal models. Collectively, these data point to a causative role of the endogenous RT as a constitutive component of an epigenetic cancer-promoting mechanism, which, when erroneously activated in differentiated cells, “resurrects” the un-differentiation program that was active in early stages of embryonic life.

### Therapeutic implications

In a recent article Hanahan [108] reviewed current anti-cancer therapies, concluding that the fourty year-lasting war against cancer, if not totally lost, has certainly not been won. In sharp contrast with current trends, he suggested to reconsider the global strategy, stepping back from the frontline of the battles against the multiple diversified features of each cancer type, and to re-approach the problem with a novel holistic view: the strategies adopted by cancer for progression, invasion and adaptive resistance should be attacked in an integrated approach and targeted simultaneously, using fewer therapeutical bullets. Ideally, the RT-targeting therapy responds to these requirements in many aspects, because:

1. RT dysregulation has a causative role in a variety of cancers by globally modulating the genome expression. As such, RT can be regarded as a common therapeutic target, acting early in cancer onset and in an ample spectrum of cancers;
2. as summarized above, RT inhibition affects the cancer transcriptome globally, reduces proliferation of cancer cells and restores their “normal” phenotypes;
3. in ongoing phase II trials on metastatic prostate carcinoma patients, RT inhibitors exert a predominant cytostatic effect with low, or no, adverse effects on patients (clinicaltrials.gov/ct2/show/NCT00964002?term=NCT00 964002&rank=1) (Piazza, unpublished results);
4. RT inhibition is sufficient to arrest, or significantly slow down, tumor progression and metastatic spreading, both in pre-clinical and small-size clinical trials, converting aggressive cancers into a chronic disease.

The RT-based therapy might therefore be suited to fulfill the requirements for a unified therapeutic approach across a large spectrum of cancer types with otherwise diversified features.

## CONCLUSIONS

Cancer has been viewed at various times as a predominantly genetic [109, 110], genomic [111, 112], epigenetic [113, 114], evolutionary [115, 116] and differentiative [103] disease. These definitions reflect the multiple perspectives under which cancer has been examined and reflect its elusive nature. In practical terms, we now view cancer as the aberrant product of genetic mutations, the consequence of epigenetic-dependent impairment of key processes, the sum of pathological alterations caused by environmental stressors or the various combinations of the above. The data discussed in this review suggest that cancer is primarily linked to what used to be called “junk DNA”, i.e. the retrotransposon component of our genome, that played crucial roles in very early stages of our development [9, 78, 79, 94, 95, 96] and is subsequently silenced and kept inactive in differentiated tissues. Cancer can therefore be viewed as the unscheduled resurrection of an embryonic mechanism within an out-of-context differentiated environment and represent the closest product to an embryo that differentiated cells can generate. In other words, LINE-1-encoded RT emerges as the tool with which cells awake the “enemy from within”.
